# Probiotic potential of lactic acid bacteria isolated from canine and feline microbiota: functional profiling and host-adapted benefits

**DOI:** 10.1007/s11259-026-11242-z

**Published:** 2026-05-04

**Authors:** Fadime Kiran, Hazal Kibar Demirhan, Sedat Sevin

**Affiliations:** 1 Pharmabiotic Technologies Research Laboratory, Department of Biology, Faculty of Science, Ankara University, Ankara, 06100 Türkiye; 2 Department of Pharmacology and Toxicology, Faculty of Veterinary Medicine, Ankara University, 06110 Ankara, Türkiye

**Keywords:** Companion animals, Probiotics, Host-adapted strains, Microbiota, Intestinal health, Immunomodulation

## Abstract

The global rise in pet ownership has increased demand for health-promoting products, particularly probiotics designed to support gastrointestinal and immune health in companion animals. However, most commercial products rely on non-host-adapted strains, which may limit gastrointestinal colonization and host-specific benefits. To address this gap, 56 bacterial isolates were obtained from the fecal and milk microbiota of clinically healthy dogs and cats. Among these, *Limosilactobacillus reuteri* DF/KS2, derived from the fecal microbiota of a Kangal Shepherd dog, and *Enterococcus faecium* CM/BS2 derived from the milk microbiota British Shorthair cat, were selected based on their broad-spectrum antimicrobial activity. Both strains exhibited a safe profile, as evidenced by γ-hemolysis and susceptibility to a panel of clinically relevant antibiotics. Under simulated gastrointestinal conditions, CM/BS2 and DF/KS2 tolerated highly acidic environments and demonstrated resilience against digestive enzymes and bile salts. Furthermore, both isolates displayed strong auto-aggregation and co-aggregation abilities with key pathogens, while adhesion assays using Caco-2 cells confirmed their capacity to inhibit pathogen attachment. Immunomodulatory evaluations further revealed that both strains effectively reduced pro-inflammatory cytokines (TNF-α, IL-1β, IL-6) and enhanced anti-inflammatory IL-10 production in canine and feline macrophages. Optimal growth occurred at 37 °C after 24 h in 2% molasses medium, and shelf-life studies demonstrated that freeze-dried cultures retained high viability over six months at − 20 °C. Collectively, these findings highlight the probiotic potential of host-adapted *L*. *reuteri* DF/KS2 and *E*. *faecium* CM/BS2, emphasizing their suitability for inclusion in species-specific probiotic formulations aimed at supporting gastrointestinal and immune health in dogs and cats.

## Introduction

According to the Global Pet Parent Study (2024), the global pet population is approaching one billion, with more than half of the world’s population reportedly owning at least one pet. Consequently, this expanding population has driven a growing demand for nutritionally balanced and functional food products designed to support overall animal health, thereby fueling the continuous growth of the pet food industry (Euromonitor International 2023). In parallel, evolving attitudes toward animal welfare, increasing awareness of species-appropriate nutrition, and a stronger emphasis on preventive veterinary care have collectively stimulated interest in health-oriented supplements, particularly probiotics (Wilson and Swanson 2024), which are defined as “live microorganisms that confer health benefits on the host when administered in adequate amounts” (Hill et al. 2014). Accordingly, the adoption of probiotic-enriched products underscores the necessity of scientifically validated strains that can survive gastrointestinal conditions and consistently confer beneficial effects in the host.

In companion animals, the gut microbiota plays a central role in maintaining intestinal homeostasis, modulating immune responses, and preventing pathogen colonization (Pujari and Banerjee 2021, Shah et al. 2024). However, external factors such as antibiotic exposure, dietary changes, and pathogen challenges can disrupt microbial balance, leading to dysbiosis and increased susceptibility to gastrointestinal disorders, including inflammatory bowel disease, ulcerative colitis, and Crohn’s disease, which are frequently observed in dogs and cats (Stavroulaki et al. 2023; Vazquez-Baeza et al. 2016; Ziese and Suchodolski 2021). Consequently, probiotics have increasingly been employed to restore microbial balance and support host health (Wilson and Swanson 2024). Importantly, the efficacy of these interventions depends not only on general probiotic properties but also on host-derived factors such as species, breed, gastrointestinal transit time, and mucosal surface area, which can influence colonization, persistence, and ultimately therapeutic outcomes (Grzeskowiak et al. 2015; Lee et al. 2022). Therefore, identifying and characterizing safe, host-adapted, and functionally robust probiotic strains remains essential for developing targeted interventions that provide reproducible, species-specific health benefits, particularly in companion animals.

Building on these considerations, the present study aimed to isolate and characterize novel lactic acid bacteria (LAB) strains from canine and feline microbiota. Beyond general probiotic traits, the functional potential of these isolates including immunomodulatory activities was evaluated using macrophages stimulated with *Escherichia coli* lipopolysaccharide (LPS). In addition, the effects of growth conditions on bacterial viability and lyophilization on shelf-life stability were assessed to determine optimal conditions for maintaining long-term probiotic functionality. Collectively, this work provides a comprehensive assessment of host-adapted LAB strains with potential applications in companion animal health, addressing both scientific and practical needs in the rapidly evolving pet food market.

## Materials and methods

### Bacterial strains and growth conditions

In this study, methicillin-resistant *Staphylococcus aureus* (MRSA) ATCC 43300, *Escherichia coli* O157:H7 ATCC 700728, *Pseudomonas aeruginosa* ATCC 35032, and *Salmonella enterica* subsp. *enterica* serovar Typhimurium ATCC 14028 were used as the indicator pathogenic bacteria. All pathogens were cultured in TSB (Tryptic Soy Broth) at 37 °C for 24 h under aerobic conditions. Unless otherwise specified, all media, chemicals, and reagents were purchased from Merck (Darmstadt, Germany) or Sigma-Aldrich (St. Louis, MO, USA).

### Sample collection

Milk samples and rectal swabs were collected from clinically healthy domestic cats and dogs by a licensed veterinarian at pet hospitals and clinics during routine medical examinations or vaccination visits. All procedures were approved by the Ankara University Institutional Animal Care and Use Committee (protocol number: 2017-4-29), and informed consent was obtained from all pet owners. Specifically, milk samples were aseptically collected from female cats (Tabby, British Shorthair, Turkish Angora, Scottish Fold; *n* = 2) and dogs (Kangal Shepherd, Pomeranian, Jack Russell Terrier, Golden Retriever; *n* = 2). Prior to sampling, the nipples and surrounding skin were carefully disinfected with 70% ethanol for 30 s and rinsed with sterile saline. The first 2–3 drops of milk were discarded to minimize contamination, and the remaining milk was collected into sterile 2mL tubes and immediately placed on ice. In addition, rectal swabs were collected from 40 healthy animals under six month of age (20 dogs and 20 cats). Dog breeds included Jack Russell Terrier, Kangal Shepherd, German Shepherd, Pekingese, and Golden Retriever (*n* = 4), while cat breeds included Tabby, British Shorthair, Turkish Angora, Scottish Fold, and Persian (*n* = 4). Sterile swabs were inoculated into pre-reduced MRS broth supplemented with 0.05% L-cysteine and transported under cold-chain conditions to the laboratory within 2 h for microbiological analysis.

### **Isolation**,** purification**,** and preservation of LAB**

Milk samples (0.5 mL) were inoculated into 9.5 mL of MRS broth and incubated under aerobic conditions at 37 °C for 48 h. Cultures were then serially diluted using sterile phosphate-buffered saline (PBS) and pour-plated onto MRS agar, followed by incubation at 37 °C for 24–48 h. Swab samples were processed using the same procedure after pre-enrichment in MRS broth. After incubation, colonies exhibiting smooth morphology and white or milky white appearance were selected and purified by streak plating on MRS agar (Sevin et al. 2021). Preliminary identification was performed using conventional methods based on Bergey’s Manual of Determinative Bacteriology, including Gram staining, colony morphology, and catalase testing (Bergey and Holt 2000). All isolates were preserved in 50% glycerol (1:1, v/v) at − 80 °C and activated by three successive subcultures prior to use.

### Identification of isolates

Identification of the isolates was performed using Matrix-Assisted Laser Desorption/Ionization Time-of-Flight Mass Spectrometry (MALDI-TOF MS), followed by molecular identification based on 16 S rRNA gene sequencing. For MALDI-TOF MS analysis, a fresh bacterial colony was applied onto a MALDI target plate, overlaid with matrix solution, and air-dried. Spectra were acquired using a calibrated MALDI-TOF MS instrument (Bruker Daltonics, Germany), and identification was carried out by comparing the generated protein spectra with the reference database provided by the system. For sequencing the 16 S rRNA gene, genomic DNA was extracted using a commercial kit (Qiagen, Germany), and PCR amplification was conducted using universal primers 27 F (5′-AGAGTTTGATCCTGGCTCAG-3′) and 1492R (5′-GGTTACCTTGTTACGACTT-3′) (Lane 1991) on a Thermal Cycler (Bio-Rad, USA). PCR reactions (50 µL) contained 10× Taq buffer (5 µL), PCR dye (5 µL), dNTPs (1 µL), Taq polymerase (0.5 µL), template DNA (2 µL), and primers (2 µL each). Cycling conditions included initial denaturation at 95 °C for 5 min, followed by 30 cycles of denaturation (95 °C, 30 s), annealing (55 °C, 30 s), and extension (72 °C, 90 s), with a final extension at 72 °C for 10 min. PCR products were analyzed on 1% agarose gels, purified using a gel extraction kit (Qiagen), and sequenced by BM Labosis Inc. (Ankara, Türkiye). Sequence homology was assessed by aligning the obtained sequences against the database of National Center for Biotechnology Information (NCBI) using the Basic Local Alignment Search Tool (BLAST).

### Screening for antimicrobial activity

Antimicrobial activity was evaluated using the disc diffusion method (Savadogo et al. 2004). Agar plates were overlaid with TSB soft agar containing pathogenic strains adjusted to 0.5 McFarland standard. Sterile paper discs (6 mm) were placed on the agar surface and inoculated with 10 µL of LAB suspensions adjusted to the same turbidity. Plates were incubated at 37 °C for 24 h, and inhibition zones were measured in millimeters. All experiments were performed in triplicate. Inhibition levels were classified as high (> 15 mm), moderate (10–15 mm), low (< 10 mm), or absent (Foongsawat et al. 2023). Isolates exhibiting strong inhibition (> 15 mm) against at least three pathogens were selected for further analyses.

### Safety assessment of selected isolates

Hemolytic activity was assessed on Columbia agar supplemented with 5% sheep blood (Oxoid, UK) following Lombardi et al. (2004). Each isolate was streaked onto agar plates, incubated at 37 °C for 48 h and examined for α-, β-, or γ-hemolysis.

Antibiotic susceptibility was determined against nine antibiotics (kanamycin, ampicillin, vancomycin, streptomycin, gentamicin, erythromycin, tetracycline, chloramphenicol, and clindamycin) using the Kirby–Bauer disc diffusion method (Bauer et al. 1959) in accordance with EFSA guidelines (EFSA 2012). Overnight cultures adjusted to 0.5 McFarland were spread on Mueller–Hinton agar and incubated at 37 °C for 24 h. Susceptibility was interpreted as resistant (R), intermediate (I), or sensitive (S) based on Clinical and Laboratory Standards Institute (CLSI) criteria (El-Shaer et al. 2017). Minimum inhibitory concentrations (MIC) were additionally determined using microtiter assays following CLSI protocols (CLSI 2012). The susceptibility results were interpreted according to EFSA (2012) microbial cut-off values.

## Survivability under gastrointestinal stress conditions

Tolerance to gastrointestinal stress was evaluated by exposing isolates to acidic pH, digestive enzymes, and bile salts. For acid tolerance, MRS broth was adjusted to pH 2 and pH 3, with pH 6.5 as control. Overnight cultures (≈ 10⁷ CFU/mL) were incubated at 37 °C for 2 h. Enzyme tolerance was tested in MRS broth adjusted to pH 2 and pH 3 containing pepsin (3 mg/mL) and pancreatin (1 mg/mL). Bile tolerance was assessed in MRS broth supplemented with 0.3%, 0.5%, and 1% oxgall. After incubation, viable counts were determined by plating serial dilutions on MRS agar. Survival rates were calculated according to Maragkoudakis et al. (2006).

## Auto-aggregation and co-aggregation assays

Auto-aggregation and co-aggregation capacity of LAB isolates were evaluated based on the method described by Chantanawilas et al. (2024). For auto-aggregation, overnight cultures of the isolates were harvested by centrifugation at 5,000x*g* for 15 min, washed twice with PBS, and resuspended to a final concentration of approximately 10^8^ CFU/mL. The suspensions were vortexed for 10 s and incubated at 37 °C for 5 h. Auto-aggregation was quantified using the following formula, where *A₀* is the optical density at 600_nm_ at time 0 and *Aₜ* is the optical density at a given time point:

$$Auto-aggregation\;(\%)=(1-A_t/A_0)\times100$$

To assess co-aggregation activity, bacterial suspensions were prepared as described above and mixed in equal volumes with suspensions of selected pathogens. The mixtures were incubated at 37 °C for 5 h, and absorbance was measured at 600_nm_. Co-aggregation was calculated by comparing the OD values of mixed and individual suspensions, using the following formula, where *Aiₛₒₗₐₜₑ* and *Aₚₐₜₕₒ*_*g*_*ₑₙ* represent the OD₆₀₀ values of individual LAB and pathogen suspensions, respectively, and *Aₘiₓ* represents that of the mixture:

$$\begin{array}{c}Co-aggregation\;(\%)=\lbrack(A_{isolate}+A_{pathogen})\\/2-A_{mix}/(A_{isolate}+A_{pathogen})/2\;\rbrack\times100\end{array}$$

### Adhesion assays and inhibition of pathogen binding to Caco-2 cells

The human epithelial colorectal adenocarcinoma cell line Caco-2 (ATCC HTB-37) were cultured in RPMI-1640 (Gibco, USA) medium supplemented with 10% fetal bovine serum (FBS) and 1× penicillin–streptomycin and incubated at 37 °C in a humidified atmosphere containing 5% CO_2_. Upon reaching approximately 80% confluence, the cells were washed with 1×PBS, detached using 0.25% trypsin–EDTA (Sartorius, Germany), and centrifuged at 200×*g* for 5 min. Cell viability was assessed using trypan blue staining and counted using a TC20 automated cell counter (Bio-Rad, USA).

For adhesion assays, the multiplicity of infection (MOI) was standardized to 100:1 (bacteria to Caco-2 cells) (Carasi et al. 2015), and cytotoxicity was first evaluated at MOIs of 10:1, 50:1, and 100:1 using the MTT assay (Mosmann 1983). Non-cytotoxic concentration (≥ 90% cell viability) was selected and co-incubated with Caco-2 cells (1 × 10⁵ cells/mL) at 37 °C in a humidified atmosphere with 5% CO_2_ for 4 h. After incubation, the culture medium was removed, and the wells were washed twice with sterile PBS to eliminate non-adherent cells. To detach the adhered cells, the Caco-2 monolayers were treated with 1% Triton X-100. The lysates were subsequently centrifuged, and the resulting pellets were resuspended in 1mL of PBS. To determine the adhered bacterial cells (CFU/mL), serial dilutions of the suspensions were plated on MRS agar and incubated at 37 °C for 48–72 h. Control wells containing an equal volume and concentration of LAB in the absence of Caco-2 cells were included to account for potential bacterial growth during the 4 h incubation. The adhesion rate was then calculated using the following formula, where *N* is the number of adherent bacteria and *N₀* is the initial number of bacteria added to the wells (Maragkoudakis et al. 2006).

$$Adhesion\;rate\;(\%)=(N/N_0)\times100$$

For pathogen inhibition assays, Caco-2 monolayers were first incubated with LAB isolates at selected MOI concentration for 4 h, followed by addition of pathogen suspensions (10⁸ CFU/mL) for 90 min. Following incubation, Caco-2 cells were lysed using 1% Triton X-100, and the number of adhered pathogenic bacteria was quantified by plating on MacConkey agar and incubating at 37 °C for 24 h The inhibition of pathogen adhesion was evaluated by comparing the number of adhered pathogens in LAB-treated wells versus untreated controls (Maragkoudakis et al. 2006).

### DPPH radical scavenging activity

The DPPH (2,2-diphenyl-1-picrylhydrazyl) free radical scavenging activity of LAB isolates was evaluated according to the method described by Das and Goyal (2015). Briefly, bacterial suspensions (10⁹ CFU/mL) were mixed with DPPH solution (0.4 mM in methanol) and incubated in the dark for 30 min. After centrifugation at 8,000×*g* for 10 min, absorbance was measured at 517_nm_. Ascorbic acid was used as a positive control. Radical scavenging activity was calculated using the following equation, where *A*_*sample*_ is the absorbance of the mixture of bacterial cells and DPPH solution, *A*_*blank*_ is the absorbance of the bacterial suspension mixed with methanol (without DPPH), and *A*_*control*_ is the absorbance of the DPPH solution alone.

$$\begin{array}{c}DPPH\;scavenging\;activity\;(\%)=\\1-\left(A_{sample}-A_{blank}/A_{control}\right)\times100\end{array}$$

### Immunomodulatory activity

Immunomodulatory effects of LAB isolates were evaluated using the canine macrophage cell line DH82 (ATCC CRL-10389) and the feline macrophage cell line Fcwf-4 (ATCC CRL-2787). Both cell lines were cultured in Dulbecco’s Modified Eagle Medium (DMEM) supplemented with 10% FBS, 2 mM L-glutamine, and non-essential amino acid solution. DH82 and Fcwf-4 cells were seeded at a density of 5 × 10^3^ cells per well in 96-well plates and incubated at 37 °C in a humidified atmosphere with 5% CO_2_ until approximately 80% confluency was reached. Prior to immunomodulatory assays, bacterial suspensions were prepared at MOI of 10:1, 50:1, and 100:1 as described before, and co-incubated with macrophage cells (1 × 10⁵ cells/mL). After a 24 h incubation period, cell viability was assessed using the MTT assay. Non-cytotoxic concentrations of MOI levels were used for subsequent immunomodulatory assays.

To further evaluate the immunomodulatory effects, macrophage cells were treated with bacterial suspensions either in the presence or absence of *Escherichia coli*-derived lipopolysaccharide (LPS, 1 µg/mL). After 24 h of stimulation, culture supernatants were collected for analysis (Quilodran-Vega et al. 2023). Nitric oxide (NO) production was measured using the Griess assay (Robbins et al. 2006), and cytokine levels (IL-6, IL-1β, IL-8, TNF-α) were quantified using ELISA kits according to the manufacturer’s instructions.

### Optimization of growth conditions and shelf-life evaluation

To identify cost-effective growth conditions, LAB isolates were cultured in alternative media formulated with whey (1–20%) and molasses (1–3%). Whey was obtained from the Dairy Technology Pilot Plant at Ankara University (Turkiye), while food-grade molasses was purchased from a local commercial supplier (Ankara, Turkiye). MRS broth was used as positive control for growth performance comparisons. Cultures were incubated at various temperatures (30, 35, and 37 °C) for 12, 24, 36, and 48 h. Growth was evaluated by viable counts on MRS agar.

For shelf-life studies, cultures were harvested, washed, and resuspended in a cryoprotectant solution containing 10% skim milk and 5% sucrose before lyophilization. Freeze-dried samples were stored at room temperature, 4 °C, and − 20 °C. Viable cell counts (CFU/mL) were determined immediately after lyophilization (day 0) and at 1st, 2nd, and 3rd months of storage by plating on MRS agar.

### Statistical analyses

All experiments were performed in triplicate and independently repeated three times. Data are presented as means ± standard deviation (SD). Prior to statistical analyses, the normality of each dataset was assessed using the Shapiro–Wilk test. For normally distributed data, differences between groups were evaluated using One-way or Two-way analysis of variance (ANOVA), followed by appropriate post-hoc tests (Dunnett, Tukey’s, or Bonferroni) to determine significant differences. Non-normally distributed data were analyzed using the Kruskal–Wallis test as appropriate. A p-value of ≤ 0.05 was considered statistically significant. All analyses were conducted using GraphPad Prism version 6.0 software.

## Results

### Selection and molecular identification of functionally promising LAB isolates

**Fig. 1:**
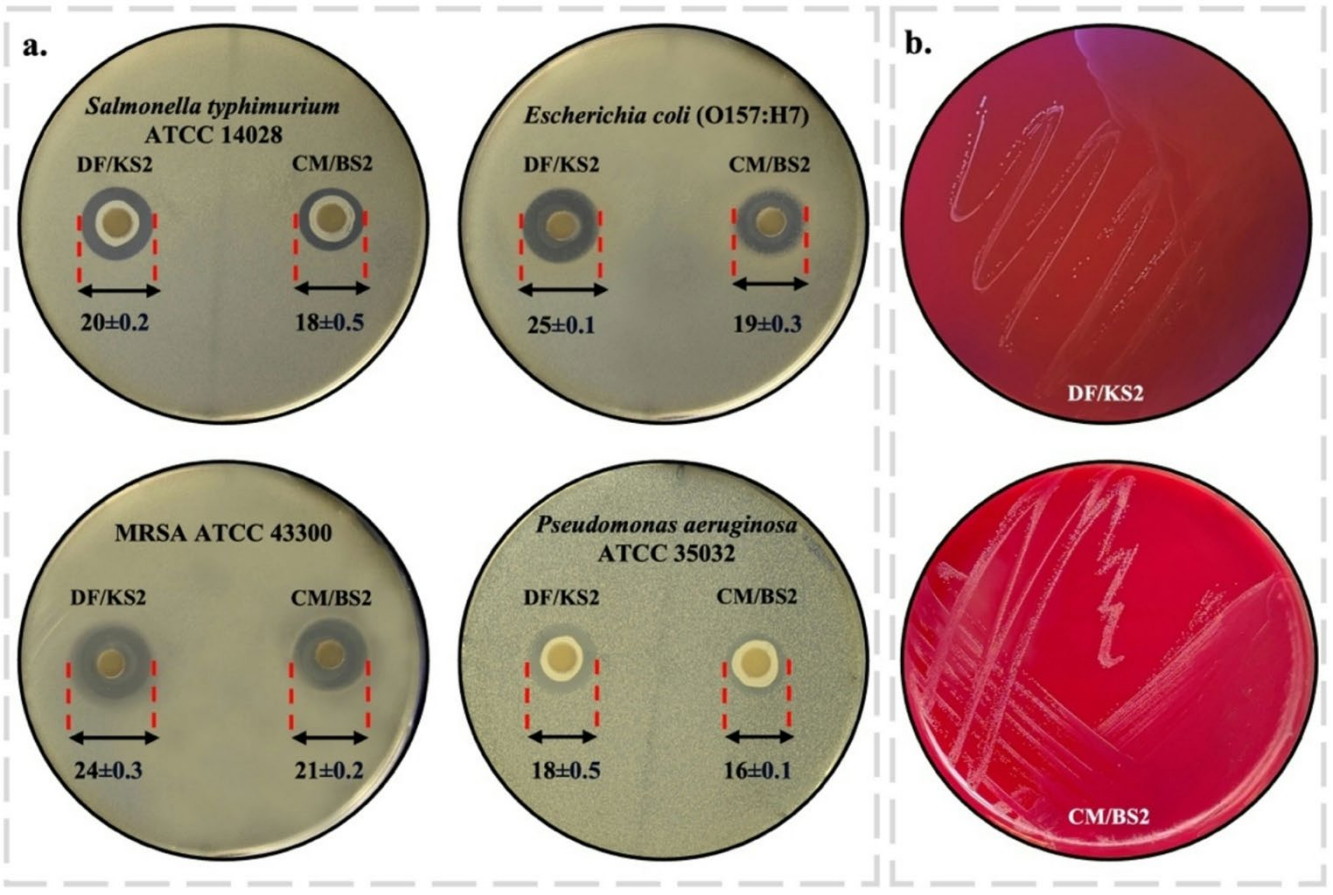
(**a**) Antimicrobial activity of selected bacterial isolates determined by disc diffusion assay (mm); (**b**) Hemolytic activities on sheep blood agar

A total of 56 LAB isolates were obtained from 16 milk samples and 40 fecal swabs collected from clinically healthy dogs and cats (Table 1). One colony per sample was selected based on typical morphological characteristics (2–3 mm diameter, pale white to yellow, translucent appearance). Microscopic examination revealed that all isolates were Gram-positive, non–endospore-forming bacteria with short rod or coccus morphology and catalase-negative activity. Initial antimicrobial screening showed variable inhibitory activity against the tested pathogens, with inhibition zones ranging from 0 to 25 mm (Table 1). Among the isolates, 26 exhibited no detectable inhibition, whereas 22 displayed weak activity (< 10 mm) against one or two pathogens. Overall, isolates derived from canine samples tended to exhibit stronger antimicrobial activity than those obtained from feline sources. Notably, only eight isolates inhibited all tested pathogens. Among these, two isolates demonstrated particularly strong and broad-spectrum activity (> 15 mm): a Gram-positive coccus isolated from the milk microbiota of a British Shorthair cat (CM/BS2) and a Gram-positive rod isolated from the gut microbiota of a Kangal Shepherd dog (DF/KS2) (Fig. 1a). These two isolates were therefore selected for further characterization. The selected isolates were preliminary identified by MALDI-TOF MS, which revealed that isolate DF/KS2 most closely matched *Limosilactobacillus reuteri* with a score value of 2.612, corresponding to a high-confidence species-level identification (score ≥ 2.0), while isolate CM/BS2 was identified as *Enterococcus faecium* with a score value of 2.412, also supporting a reliable species-level assignment. To confirm these results, molecular identification was further performed by sequencing the 16 S rRNA gene region (~ 1,500 bp), followed by BLAST analysis against the GenBank database. Based on sequence similarity, isolate DF/KS2 showed 98% identity to *Limosilactobacillus reuteri*, whereas isolate CM/BS2 exhibited 99% identity to *Enterococcus faecium*. The sequences were deposited in the NCBI database under accession numbers PV247127.1 for *Limosilactobacillus reuteri* DF/KS2 and PV247097.1 for *Enterococcus faecium* CM/BS2.

**Table 1:** Antimicrobial activity of LAB isolates

Samples/Breeds	Numbers (*n*)	Selected colony codes	Antimicrobial activity (Inhibition zones: mm)			
			MRSA ATCC 43300	*E. coli* O157:H7	*P*. aeruginosa ATCC 35032	*S.* Typhimurium ATCC 14028
**Milk samples (M)**						
**CATS (C)**						
Tabby (T)	2 ♀	CM/T1 CM/T2	- -	- -	- -	- -
British Shorthair (BS)	2 ♀	CM/BS1 **CM/BS2**	12 ± 0.3 21 ± 0.2	10 ± 0.1 19 ± 0.3	7 ± 0.1 16 ± 0.1	10 ± 0.6 18 ± 05
Turkish Angora (TA)	2 ♀	CM/TA1 CM/TA2	- 6 ± 0.1	- 6 ± 0.2	- -	- -
Scottish Fold (SF)	2 ♀	CM/SF1 CM/SF2	8 ± 0.4 -	- -	- -	- -
**DOGS (D)**						
Kangal Shepherd (KS)	2 ♀	DM/KS1 DM/KS2	10 ± 0.5 -	11 ± 0.1 -	8 ± 0.1 -	11 ± 0.3 -
Pomeranian (P)	2 ♀	DM/P1 DM/P2	- -	- -	- -	- -
Jack Russell Terrier (JRT)	2 ♀	DM/JRT1 DM/JRT2	- 9 ± 0.2	- 10 ± 0.2	- 7 ± 0.2	- 12 ± 0.4
Golden (G)	2 ♀	DM/G1 DM/G2	10 ± 0.6 -	11 ± 0.7 -	7 ± 0.2 -	11 ± 0.1 -
**Fecal samples (F)**						
**CATS (C)**						
Tabby (T)	2 ♀ / 2 **♂**	CF/T1 CF/T2 CF/T3 CF/T4	- 6 ± 0.4 7 ± 0.3 -	- - 7 ± 0.1 -	- - - -	- - - -
British Shorthair (BS)	2 ♀ / 2 **♂**	CF/BS1 CF/BS2 CF/BS3 CF/BS4	9 ± 0.4 8 ± 0.1 - -	9 ± 0.5 - - -	6 ± 0.5 5 ± 0.4 - -	8 ± 0.2 - - -
Turkish Angora (TA)	2 ♀ / 2 **♂**	CF/TA1 CF/TA2 CF/TA3 CF/TA4	- - - 7 ± 0.2	- - - 5 ± 0.6	- - - -	8 ± 0.7 - - -
Scottish Fold (SF)	2 ♀ / 2 **♂**	CF/SF1 CF/SF2 CF/SF3 CF/SF4	6 ± 0.3 6 ± 0.1 - -	7 ± 0.3 6 ± 0.5 - -	- - - -	- - - -
Persian (P)	2 ♀ / 2 **♂**	CF/P1 CF/P2 CF/P3 CF/P4	- - - 7 ± 0.2	- - - -	- - - -	- - - 7 ± 0.3
**DOGS (D)**						
Jack Russell Terrier (JRT)	2 ♀ / 2 **♂**	DF/JRT1 DF/JRT2 DF/JRT3 DF/JRT4	9 ± 0.1 - 8 ± 0.3 -	8 ± 0.4 - 7 ± 0.5 -	- - - -	- - - -
Kangal Shepherd (KS)	2 ♀ / 2 **♂**	DF/KS1 **DF/KS2** DF/KS3 DF/KS4	13 ± 0.1 24 ± 0.3 8 ± 0.5 9 ± 0.4	12 ± 0.1 25 ± 0.1 - -	9 ± 0.4 18 ± 0.5 - -	11 ± 0.2 20 ± 0.2 8 ± 0.1 -
German Shepherd (GS)	2 ♀ / 2 **♂**	DF/GS1 DF/GS2 DF/GS3 DF/GS4	- 7 ± 0.1 - -	- 6 ± 0.5 - -	- - 6 ± 0.2 -	- - - -
Pekingese (P)	2 ♀ / 2 **♂**	DF/P1 DF/P2 DF/P3 DF/P4	- - 7 ± 0.3 -	8 ± 0.2 - - -	8 ± 0.4 - 7 ± 0.3 5 ± 0.1	- - - -
Golden Retriever (GR)	2 ♀ / 2 **♂**	DF/GR1 DF/GR3 DF/GR1 DF/GR4	9 ± 0.4 - - 8 ± 0.2	- - 7 ± 0.2 -	- - 8 ± 0.3 -	9 ± 0.1 - - 7 ± 0.6

### Safety profile of LAB strains: Hemolytic potential and antibiotic susceptibility

The safety profiles of selected strains (*L. reuteri* DF/KS2 and *E. faecium* CM/BS2) were assessed through hemolytic activity and antibiotic susceptibility tests. Neither isolate exhibited hemolytic activity on sheep blood agar, as evidenced by the absence of clear or greenish zones surrounding colonies, confirming γ-hemolysis (non-hemolytic) phenotype (Fig. 1b). In terms of antibiotic susceptibility, both isolates were tested against nine clinically relevant antibiotics using the disc diffusion and MIC assays in accordance with EFSA (2012) and CLSI (2012, 2020) guidelines. Both strains demonstrated susceptibility to all tested antibiotics, with inhibition zones and MIC values falling within acceptable EFSA-recommended cut-off limits (Table 2).

**Table 2:** Antibiotic susceptibility of the selected LAB strains

Antibiotics	Disc concentrations/ Inhibition zones	Susceptibility Cut-off (CLSI, 2017, 2020)			MIC (mg/L)	Cut-off values (mg/L) (EFSA,^2012^)	Results
		*R*	I	S			
***L. reuteri *** **DF/KS2**							
**Ampicillin**	10 µg / 28 ± 0.6 mm	≤ 13	13–14	≥ 15	0.5	2	**S**
**Vancomycin**	30 µg / 0 mm	≤ 14	15–16	≥ 17	n.r.	n.r.	**n.r.**
**Gentamicin**	10 µg / 17 ± 0.1 mm	≤ 12	13–14	≥ 15	4	8	**S**
**Kanamycin**	30 µg / 20 ± 0.6 mm	≤ 13	14–17	≥ 18	32	64	**S**
**Streptomycin**	10 µg / 17 ± 0.2 mm	≤ 11	13–16	≥ 17	32	64	**S**
**Erythromycin**	10 µg / 33 ± 0.6 mm	≤ 13	14–22	≥ 23	0.5	1	**S**
**Clindamycin**	10 µg / 39 ± 0.8 mm	≤ 15	16–20	≥ 21	0.5	1	**S**
**Tetracycline**	30 µg / 28 ± 0.6 mm	≤ 14	15–18	≥ 18	4	16	**S**
**Chloramphenicol**	30 µg / 34 ± 0.8 mm	≤ 12	13–17	≥ 18	2	4	**S**
***E. faecium*** **CM/BS2**							
**Ampicillin**	10 µg / 32 ± 0.4 mm	≤ 16	-	≥ 17	1	2	**S**
**Vancomycin**	30 µg / 23 ± 0.7 mm	≤ 14	15–16	≥ 17	2	4	**S**
**Gentamicin**	120 µg / 14 ± 0.2 mm	≤ 6	7–9	≥ 10	4	32	**S**
**Kanamycin**	30 µg / 20 ± 0.4 mm	≤ 13	14–17	≥ 18	32	1024	**S**
**Streptomycin**	300 µg / 19 ± 0.5 mm	≤ 6	7–9	≥ 10	32	128	**S**
**Erythromycin**	15 µg / 30 ± 0.6 mm	≤ 13	14–22	≥ 23	1	4	**S**
**Clindamycin**	10 µg / 38 ± 0.7 mm	≤ 15	16–20	≥ 21	0.5	4	**S**
**Tetracycline**	30 µg / 22 ± 0.3 mm	≤ 14	15–18	≥ 19	2	4	**S**
**Chloramphenicol**	30 µg / 30 ± 0.6 mm	≤ 12	13–17	≥ 18	4	16	**S**

*S* Sensitive, *I* Intermediate,* R* Resistant, *n.r.* not required

### Survivability of LAB strains under simulated gastrointestinal stress conditions

**Fig. 2:**
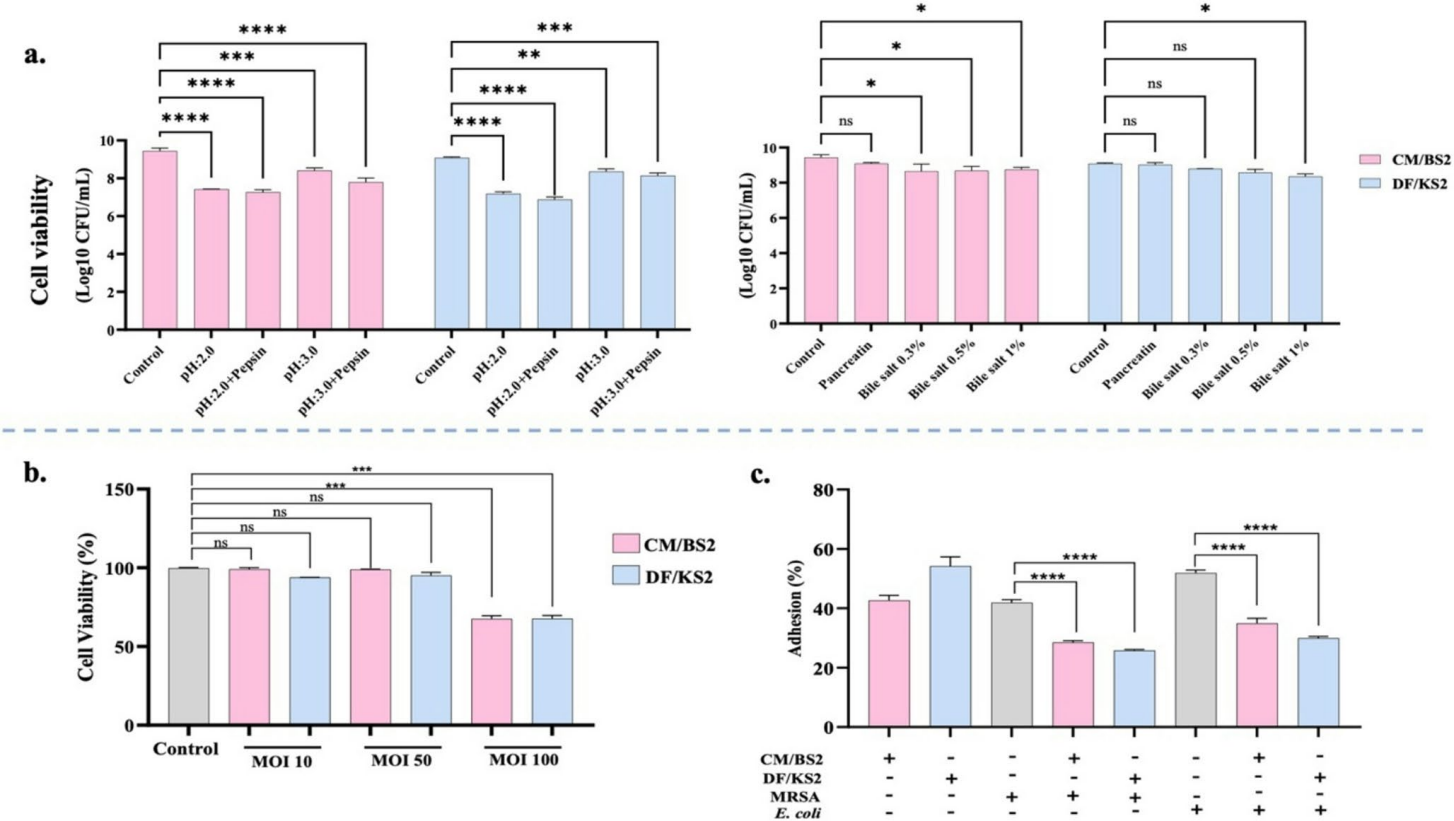
(**a**) Survival of *L. reuteri* DF/KS2 and *E. faecium* CM/BS2 under simulated gastrointestinal conditions, (**b**) Viability of Caco-2 cells at different multiplicities of infection (MOI 10:1, 50:1, and 100:1) of bacterial suspensions; (**c**) Adhesion capacities of the isolates to Caco-2 cells and inhibition of pathogen adhesion (*p < 0.05; **p < 0.01; ***p < 0.001; ****p < 0.0001; ns: non-significant)

The tolerance of *L*. *reuteri* DF/KS2 and *E*. *faecium* CM/BS2 to simulated gastrointestinal stress conditions including acidic pH, digestive enzymes, and bile salts was evaluated (Fig. 2a). Under control conditions, the initial viable counts were 2.1 ± 0.05 × 10⁹ CFU/mL for *E*. *faecium* CM/BS2 and 1.1 ± 0.01 × 10⁹ CFU/mL for *L*. *reuteri* DF/KS2. Exposure to pH 2.0 resulted in substantial reductions in viability. Counts decreased to 2.6 ± 0.11 × 10⁷ CFU/mL for *E*. *faecium* CM/BS2 (1.91-log reduction, *p* < 0.0001) and 1.8 ± 0.09 × 10⁷ CFU/mL for *L*. *reuteri* DF/KS2 (1.78-log reduction; *p* < 0.0001). In the presence of pepsin at pH 2.0, survival further declined to 1.5 ± 0.07 × 10⁷ CFU/mL (2.15-log reduction) and 9.4 ± 0.18 × 10⁶ CFU/mL (2.17-log reduction), respectively (*p* < 0.0001). In contrast, both strains exhibited improved tolerance at pH 3.0. *E*. *faecium* CM/BS2 retained 2.0 ± 0.12 × 10⁸ CFU/mL (1.02-log reduction; *p* < 0.001), whereas *L*. *reuteri* DF/KS2 maintained 1.8 ± 0.13 × 10⁸ CFU/mL (0.78-log reduction; *p* < 0.01). Pepsin exposure at pH 3.0 caused moderate additional reductions but still maintained substantial viability. Both strains also displayed high resistance to pancreatin, showing minimal reductions of 0.28 log for *E*. *faecium* CM/BS2 and 0.10 log for *L*. *reuteri* DF/KS2 (*p* > 0.05). Similarly, moderate decreases were observed under bile salt exposure. *E*. *faecium* CM/BS2 showed log reductions of 0.38, 0.46, and 0.48 at 0.3%, 0.5%, and 1.0% bile, respectively, while *L*. *reuteri* DF/KS2 exhibited reductions of 0.24, 0.33, and 0.58 under the same conditions. Overall, both strains demonstrated substantial tolerance to gastrointestinal stressors, although *E*. *faecium* CM/BS2 showed stronger acid resistance whereas *L*. *reuteri* DF/KS2 displayed slightly better tolerance to enzyme and bile exposure.

### Aggregation and co-aggregation capacities

Auto-aggregation and co-aggregation abilities were evaluated to assess the potential of the isolates to colonize intestinal surfaces and interfere with pathogen adhesion. After 5 h incubation, *L*. *reuteri* DF/KS2 exhibited a higher auto-aggregation capacity (89.78 ± 1.42%) compared with *E*. *faecium* CM/BS2 (81.15 ± 1.04%). In co-aggregation assays with MRSA ATCC 43,300 and *E*. *coli* O157:H7 ATCC 700,728, *L*. *reuteri* DF/KS2 demonstrated the highest co-aggregation ability, reaching 78.18 ± 2.34% with *E*. *coli* and 76.21 ± 3.21% with MRSA. *E*. *faecium* CM/BS2 also showed strong co-aggregation capacities, with 71.12 ± 2.78% and 65.15 ± 2.08% against *E*. *coli* and MRSA, respectively (*p* < 0.05).

### Adhesion to CaCo-2 cells and inhibition of pathogen attachment

Cytotoxicity testing indicated that MOI 10:1 and 50:1 did not significantly affect Caco-2 cell viability (*p* > 0.05), whereas MOI 100:1 significantly reduced viability (*p* < 0.001). Specifically, *L*. *reuteri* DF/KS2 and *E*. *faecium* CM/BS2 reduced viability by approximately 39 ± 0.87% and 22.3 ± 0.11%, respectively. Therefore, MOI 50:1 was selected for subsequent assays (Fig. 2b). Under these conditions, adhesion to Caco-2 cells reached 54.2 ± 1.02% for *L*. *reuteri* DF/KS2 and 42.64 ± 0.41% for *E*. *faecium* CM/BS2 (Fig. 2c). Moreover, both strains significantly reduced the adhesion of MRSA and *E*. *coli* O157:H7 to epithelial cells compared with untreated controls (*p* < 0.0001). Notably, *L*. *reuteri* DF/KS2 exhibited the strongest inhibitory effect, suggesting a superior capacity for competitive exclusion of pathogenic bacteria.

### DPPH radical scavenging activity

The DPPH radical scavenging activity of *L. reuteri* DF/KS2 and *E. faecium* CM/BS2 was assessed to determine their free radical neutralization capacity. Both strains exhibited notable activity. However, *L*. *reuteri* DF/KS2 demonstrated a significantly higher scavenging rate (66.07 ± 1.14%) than *E*. *faecium* CM/BS2 (52.06 ± 1.07%) (*p* < 0.05).

#### Modulation of inflammatory responses in macrophage models

**Fig. 3:**
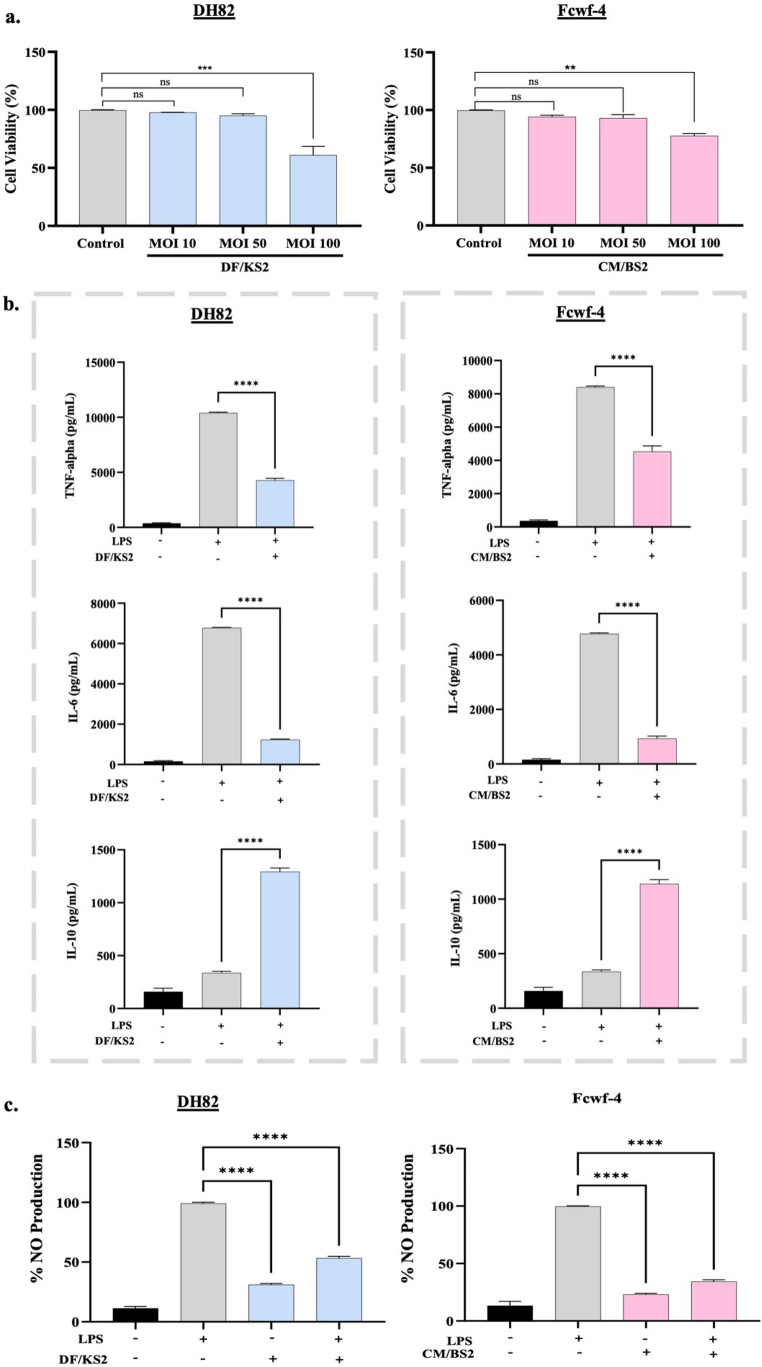
(**a**) Viability of canine (DH82) and feline (Fcwf-4) macrophage cells at different multiplicities of infection (MOI 10:1, 50:1, and 100:1) of bacterial suspensions; (**b**) Modulation of cytokine production (TNF-α, IL-6, and IL-10) in LPS-stimulated (1 µg/mL) DH82 and Fcwf-4 macrophages following co-treatment with *L. reuteri* DF/KS2 and *E. faecium* CM/BS2 (MOI 50:1); (**c**) Nitric oxide (NO) production in LPS-stimulated macrophage cells treated with same isolates (**p < 0.01; ***p < 0.001; ****p < 0.0001; ns: non-significant)

Prior to immunomodulatory assays, cytotoxicity was evaluated in canine DH82 and feline Fcwf-4 macrophage cell lines. MOI 10:1 and 50:1 maintained cell viability above 90% (*p* > 0.05), whereas MOI 100:1 significantly reduced viability (*p* < 0.01). Therefore, MOI 50:1 was selected for further experiments (Fig. 3a), and the immunomodulatory effects of the selected LAB strains were subsequently assessed by quantifying cytokine responses in DH82 and Fcwf-4 macrophages using ELISA. According to our results, both strains exhibited immunomodulatory activity in LPS-stimulated macrophages. In DH82 cells, co-treatment with *L*. *reuteri* DF/KS2 significantly reduced TNF-α (2.43-fold) and IL-6 (5.51-fold) levels compared with LPS-treated controls (*p* < 0.0001). Similarly, *E*. *faecium* CM/BS2 suppressed cytokine production in Fcwf-4 cells, reducing TNF-α (1.85-fold) and IL-6 (5.13-fold). Importantly, treatment with the LAB strains alone did not induce pro-inflammatory cytokines. Conversely, both strains significantly increased the anti-inflammatory cytokine IL-10 (*p* < 0.0001) and reduced nitric oxide production in LPS-stimulated macrophages (Fig. 3b and c).

### Optimization of cultivation parameters

**Fig. 4:**
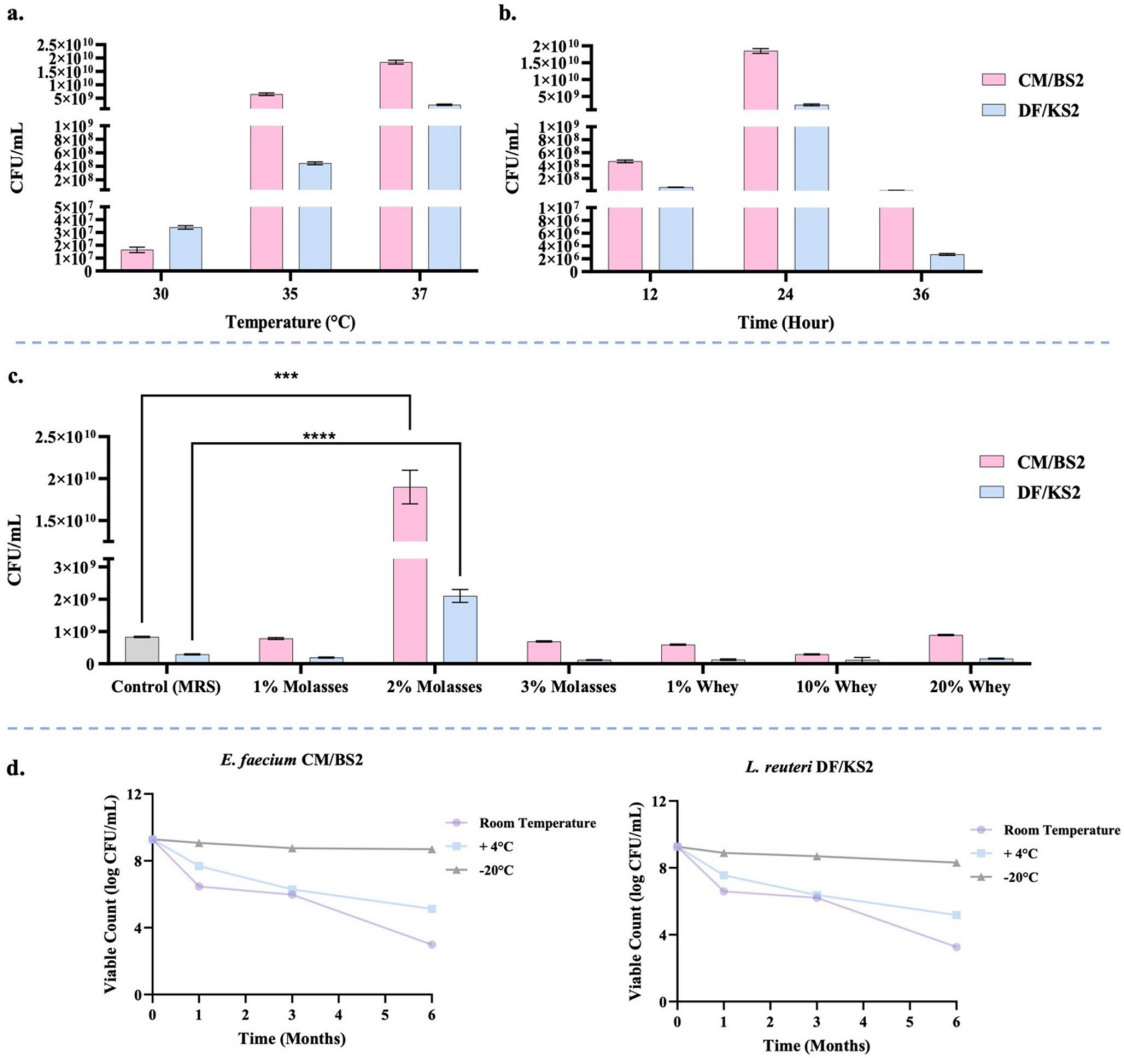
(**a**) Growth of *L. reuteri* DF/KS2 and *E. faecium* CM/BS2 at different incubation temperatures in MRS broth; (**b**) Time-dependent growth kinetics of the isolates at 37 °C, measured at 12, 24, and 36 h. (**c**) Comparative growth performance in MRS (control), and alternative media containing varying concentrations of molasses (1–3%) and whey (1–20%) after 24 h of incubation at 37 °C; (**d**) Shelf-life stability of the isolates following lyophilization and storage under different temperature conditions over a 6-month period (***p < 0.001; ****p < 0.0001)

Temperature significantly influenced bacterial growth. For *L*. *reuteri* DF/KS2, viable counts increased from 3.5 × 10⁶ CFU/mL at 30 °C to 4.1 ± 0.18 × 10⁸ CFU/mL at 35 °C and reached 2.1 ± 0.11 × 10⁹ CFU/mL at 37 °C. Similarly, *E*. *faecium* CM/BS2 increased from 1.5 ± 0.09 × 10⁷ CFU/mL at 30 °C to 6.1 ± 0.41 × 10⁹ CFU/mL at 35 °C and peaked at 1.9 ± 0.10 × 10¹⁰ CFU/mL at 37 °C (*p* < 0.001) (Fig. 4a). Statistically, 37 °C yielded significantly higher growth for both strains compared to the lower tested temperatures (*p* < 0.001), indicating that 37 °C supported the highest biomass production within the range of temperatures evaluated. At 37 °C, growth peaked after 24 h for both strains, reaching 2.1 ± 0.13 × 10⁹ CFU/mL for *L*. *reuteri* and 1.9 ± 0.15 × 10¹⁰ CFU/mL for *E*. *faecium*, after which viability declined markedly (Fig. 4b). In addition, alternative media formulations were evaluated. Among the tested conditions, 2% molasses supported the highest bacterial growth, yielding 2.1 ± 0.13 × 10⁹ CFU/mL for *L*. *reuteri* DF/KS2 and 1.9 ± 0.14 × 10^10^ CFU/mL for *E*. *faecium* CM/BS2, corresponding to significant increases compared with the MRS control (*p* < 0.001) (Fig. 4c). These findings identify 2% molasses as a cost-effective growth medium for large-scale production.

#### Shelf-life stability of LAB strains

The stability of freeze-dried LAB strains was evaluated during 6-month storage at room temperature, 4 °C, and − 20 °C. Immediately after lyophilization, viable counts decreased slightly from 10.17 to 9.25 log CFU/mL for *L*. *reuteri* DF/KS2 and from 10.30 to 9.29 log CFU/mL for *E*. *faecium* CM/BS2. Storage temperature strongly influenced viability. At room temperature, both strains showed progressive declines, reaching 3.15 and 2.99 log CFU/mL after 6 months. Storage at 4 °C resulted in moderate losses, with final counts of 5.15 and 5.04 log CFU/mL. In contrast, storage at − 20 °C provided the highest stability, maintaining counts of 8.31 and 8.60 log CFU/mL for *L*. *reuteri* DF/KS2 and *E*. *faecium* CM/BS2, respectively (Fig. 4d). Collectively, these results demonstrate that − 20 °C is the most suitable condition for preserving the long-term viability of the freeze-dried probiotic formulations.

## Discussion

Probiotics are widely utilized in functional foods and dietary supplements due to their capacity to modulate the gut microbiota and promote overall host health (Yang and Wu 2023). Although research in this field has predominantly focused on humans and livestock, investigations specifically targeting companion animals, particularly cats and dogs, remain relatively limited (Yang and Wu 2023). Nevertheless, emerging evidence demonstrated that probiotic administration in these species can improve gut microbial balance, modulate inflammation, enhance immune responses, and provide protection against infections (Karukayil Gopalakrishnan et al. 2025; Onuma et al. 2025; Rossi et al. 2018). Despite these benefits, most probiotic strains studied to date originate from non-host-adapted sources, potentially limiting their ecological fitness and functional efficiency within the target host (Melera et al. 2022). In contrast, host-derived strains are more likely to exhibit enhanced colonization, persistence, and probiotic efficacy due to evolutionary co-adaptation with their host (Jang et al. 2021; Sun et al. 2025). Given that the functional properties of probiotics are often closely linked to host specificity, it has been suggested that strains derived from the indigenous microbiota of the target host may offer the most effective and biologically compatible probiotic candidates (Rudenko et al. 2021; Yang and Wu 2023). Accordingly, the present study focused on isolating novel LAB strains from the microbiota of clinically healthy cats and dogs to identify host-adapted candidates for future probiotic applications.

The antimicrobial activity of probiotics is fundamental to host health, as inhibits pathogenic microorganisms and maintains microbial homeostasis within the gastrointestinal tract (Maftei et al. 2024). In this study, 56 LAB isolates were obtained from milk and fecal samples of clinically healthy dogs and cats, and their inhibitory effects against selected pathogens were evaluated. Notably, *E. faecium* CM/BS2, isolated from the milk microbiota of a British Shorthair cat, and *L. reuteri* DF/KS2, obtained from the fecal microbiota of a Kangal Shepherd dog, exhibited strong and consistent antimicrobial activity against all tested pathogens, which was markedly superior to the antibacterial capabilities reported for LAB in previous studies (Coman et al. 2019). These findings align with earlier reports demonstrating that specific LAB strains exert significant antagonistic effects against pathogens (Liu et al. 2024, Wang et al. 2024), further underscoring their potential for the development of host-adapted probiotic formulations. The pronounced inhibitory effects observed in probiotic systems are generally attributed to the production of antimicrobial metabolites, such as bacteriocins and other bioactive compounds, which facilitate competitive exclusion and modulate interspecies microbial dynamics within the gut (Van Zyl et al. 2020). In the present study, the observed antagonistic effects are likely associated with the acidic environment and organic acid metabolites rather than proteinaceous substances produced during active growth, as evidenced by the loss of antimicrobial activity following neutralization. Accordingly, the inhibitory activity observed herein appears to be predominantly driven by pH-dependent mechanisms and organic acid production.

Beyond their antimicrobial effects, the probiotic potential of these strains is further reinforced by their notable adhesion properties. Both isolates exhibited high auto-aggregation and co-aggregation capacities with *E. coli* and MRSA, indicating a strong ability to adhere to the intestinal epithelium and competitively exclude pathogens. Compared to previous studies, these adhesion capacities are particularly robust. For instance, a canine-derived LAB strain described by Liu et al. (2024) exhibited a maximum adhesion rate of only 3.62%, significantly lower than the rates observed in the present study. Such differences likely reflect intrinsic strain-specific characteristics and host-microbe co-evolution. Supporting this notion, Hanifeh et al. (2021) reported that canine-derived *E*. *faecalis* and *E*. *faecium* strains adhered more effectively to the intestinal mucosa of dogs than poultry-derived strains of the same species, while Kainulainen et al. (2015) found that a canine-origin *L*. *acidophilus* strain demonstrated superior adhesion to canine colonic mucus compared to porcine and human-derived strains. Taken together, the dual functionality of these LAB strains combining broad-spectrum antimicrobial activity with strong adhesion capacity highlights their potential as host-adapted biological barriers in the gastrointestinal tract, thereby enhancing mucosal defense mechanisms and effectively preventing colonization and persistence of enteric pathogens in companion animals.

Building on their demonstrated antimicrobial efficacy and adhesion capabilities, it is essential to assess the stress tolerance and safety profiles of these strains to confirm their suitability as effective and secure probiotics for companion animals. In this context, our evaluations revealed that both isolates exhibited no hemolytic activity, consistent with previous reports on LAB strains derived from canine feces (Liu et al. 2024) and feline milk (Zheng et al. 2025). Furthermore, both strains demonstrated full susceptibility to all tested antibiotics, a critical prerequisite for probiotic candidacy as outlined by EFSA guidelines (EFSA 2012). In contrast, many LAB strains may harbor antibiotic resistance genes, representing a significant safety concern (Wong et al. 2015). For instance, Kerek et al. (2024) reported that commercially available *E*. *faecium* strains for cats and dogs frequently carried multiple resistance determinants. The absence of resistance in our isolates may reflect factors such as strain origin, host species, and geographical differences (Al Kassaa et al. 2014), highlighting that antibiotic susceptibility in probiotics is both species-specific and highly strain-dependent (Ramos et al. 2013).

Regarding gastrointestinal resilience, both isolates exhibited substantial tolerance to simulated gastrointestinal stress conditions. Specifically, *E*. *faecium* CM/BS2 showed superior acid resistance, whereas *L*. *reuteri* DF/KS2 was more resilient against bile and enzymatic stress, traits that are critical for intestinal survival and colonization. Considering that the postprandial gastric pH in dogs typically exceeds 3.0 (Mahar et al. 2012), L. *reuteri* DF/KS2 is likely to survive stomach transit and maintain viability in the small intestine. Although intestinal bile concentrations in dogs and cats are not precisely defined, many isolates tolerated 0.3% bile salts, comparable to human small intestine levels (Strompfova et al. 2006). Moreover, with small intestinal transit times generally under 2 h in dogs (Iwanaga et al. 1998), these isolates are likely capable of withstanding bile stress long enough to reach and colonize the intestinal mucosa. Collectively, these findings reflect the natural adaptation of microbiota-derived strains to the host gastrointestinal environment, consistent with previous reports by Raheem et al. (2022), who observed similar acid and bile tolerance profiles in *L*. *plantarum* strain RW1 isolated from canine feces, and further underscore the importance of evaluating both physiological resilience and safety to identify robust, host-adapted probiotic candidates for companion animals.

The antioxidant properties of probiotics are increasingly recognized as a key criterion in strain selection, given that oxidative stress plays a pivotal role in the pathogenesis of metabolic, neurodegenerative, and gastrointestinal disorders (Pisoschi and Pop 2015). Consequently, identifying strains with potent antioxidant capabilities may provide additional therapeutic benefits beyond gastrointestinal health, particularly in attenuating systemic oxidative damage (Wang et al. 2024). In the present study, DPPH free radical scavenging activity of LAB isolates was evaluated. Among the tested strains, *L. reuteri* DF/KS2 exhibited the highest DPPH scavenging capacity, which is comparable to values reported for LAB strains isolated from canine feces by Zhao et al. (2023) and consistent with findings by Zhou et al. (2022). Notably, these effects gain further significance when considering the well-established link between oxidative stress and chronic inflammation (Jang et al. 2024). To explore this connection, the immunomodulatory effects of the selected LAB strains were evaluated using LPS-induced canine and feline macrophage cells. Both strains were non-cytotoxic and demonstrated beneficial effects on immune regulation, consistent with previous reports on probiotics as immunomodulators (Azad et al. 2018). In line with earlier studies demonstrating the ability of probiotics to reduce the expression of proinflammatory cytokines such as IFN-γ, IL1B, IL2, IL8, and TNF-α following pathogen-induced inflammation (Azad et al. 2018; Chen et al. 2019; Jang et al. 2024; Kainulainen et al. 2015; Souza et al. 2018), the current study revealed that *E. faecium* CM/BS2 significantly decreased IL-6 and TNF-α levels in stimulated Fcwf-4 cells. Similarly, *L. reuteri* DF/KS2 was effective in reducing the same cytokines in stimulated DH82 cells. Interestingly, although both isolates decreased the proinflammatory cytokine levels, a marked induction in IL-10 expression was also observed in immune-activated macrophages. Similar to our results, *L. rhamnosus* CACC612 and *Bifidobacterium animalis* subsp. *lactis* CACC789 isolated from a Norwegian forest cat and a Persian cat, decreased the IFN-γ, IL1β, IL2, IL4, and TNF-α levels on immune-induced Fcwf-4 macrophage cells (Jang et al. 2024). However, direct comparisons across host species remain limited. Therefore, our results provide preliminary evidence supporting the efficacy of host-adapted probiotic candidates, although further research is needed to elucidate the specific interactions between these strains and their hosts. Collectively, these data suggest that the selected strains not only possess natural antioxidant potential, particularly in feline and canine hosts, but may also contribute to the modulation of inflammatory responses, an increasingly recognized therapeutic target in probiotic research.

In conclusion, this study reports the identification and functional characterization of two novel probiotic strains, *L. reuteri* DF/KS2 and *E. faecium* CM/BS2, isolated from canine and feline microbiota. In vitro analyses demonstrated that both strains exhibit broad-spectrum antimicrobial activity, notable antioxidant potential, immunomodulatory effects, and strong tolerance to gastrointestinal stressors, collectively underscoring their promise as host-adapted probiotics for companion animals. Furthermore, their ability to grow in low-cost media and retain high viability under cold-chain storage conditions enhances their feasibility for commercial application. Although these in vitro findings provide a solid foundation, in vivo studies are essential to confirm their colonization capacity, safety, and efficacy within the gastrointestinal tracts of canine and feline hosts. Taken together, these results suggest that the selected strains hold substantial potential not only for supporting gut and immune health in pets but also for facilitating the development of species-specific probiotic formulations tailored to the unique physiological requirements of companion animals.

## Data Availability

The datasets of 16 S rRNA gene sequencing generated during the current study have been deposited in the NCBI GenBank repository under the accession numbers PV247127.1 ( *Limosilactobacillus reuteri* DF/KS2) and PV247097.1 ( *Enterococcus faecium* CM/BS2). Other data supporting the findings of this study are available from the corresponding author upon reasonable request.
